# Interleukin-32 in systemic sclerosis, a potential new biomarker for pulmonary arterial hypertension

**DOI:** 10.1186/s13075-020-02218-8

**Published:** 2020-06-01

**Authors:** Paola Di Benedetto, Giuliana Guggino, Giovanna Manzi, Piero Ruscitti, Onorina Berardicurti, Noemi Panzera, Nicolò Grazia, Roberto Badagliacca, Valeria Riccieri, Carmine Dario Vizza, Ganna Radchenko, Vasiliki Liakouli, Francesco Ciccia, Paola Cipriani, Roberto Giacomelli

**Affiliations:** 1 Clinical Pathology Unit, Department of Biotechnological and Applied Clinical Sciences, University of L’Aquila, L’Aquila, Italy; 2 Rheumatology Section, Department of Internal Medicine, University of Palermo, Palermo, Italy; 3 Department of Cardiovascular and Respiratory Sciences, Sapienza University of Rome, Rome, Italy; 4 Division of Rheumatology, Department of Biotechnological and Applied Clinical Sciences, University of L’Aquila, L’Aquila, Italy; 5 Department of Internal Medicine and Medical Specialities, Sapienza University of Rome, Rome, Italy; 6 Secondary Hypertension Department with Pulmonary Hypertension Center, State Institute National Scientific Center “MD Strazhesko Institute of Cardiology” of Ukrainian National Academy of Medical Science, Kyiv, Ukraine; 7 Rheumatology Section, Department of Clinical and Experimental Medicine, University of Campania “Luigi Vanvitelli”, Naples, Italy

**Keywords:** Systemic sclerosis, Pulmonary arterial hypertension, IL-32

## Abstract

**Background:**

Pulmonary arterial hypertension (PAH) is a severe complication of systemic sclerosis (SSc), associated with a progressive elevation in pulmonary vascular resistance and subsequent right heart failure and death. Due to unspecific symptoms, the diagnosis of PAH is often delayed. On this basis, it is of great value to improve current diagnostic methods and develop new strategies for evaluating patients with suspected PAH. Interleukin-32 (IL-32) is a proinflammatory cytokine expressed in damaged vascular cells, and the present study aimed to assess if this cytokine could be a new biomarker of PAH during SSc.

**Methods:**

The IL-32 expression was evaluated in the sera and skin samples of 18 SSc-PAH patients, 21 SSc patients without PAH, 15 patients with idiopathic PAH (iPAH) and 14 healthy controls (HCs), by enzyme-linked immunosorbent assay (ELISA) and immunohistochemistry (IHC). Receiver-operating characteristic (ROC) curves were performed to evaluate the cut-off of IL-32 in identifying patients with PAH. Furthermore, in SSc patients, correlation analyses were performed between IL-32 sera levels and mean pulmonary artery pressure (mPAP) evaluated by right heart catheterization (RHC) and systolic pulmonary artery pressure (sPAP), obtained by echocardiography. Additionally, the number of skin IL-32+ cells was correlated with modified Rodnan skin score (mRSS).

**Results:**

In SSc-PAH patients, IL-32 sera levels were significantly higher when compared with SSc patients without PAH and patients affected by iPAH. The analysis of ROC curve showed that IL-32 sera levels above 11.12 pg/ml were able to predict patients with PAH (sensitivity = 90%, specificity = 100%). Furthermore, the IL-32 sera levels of patients with SSc correlated with both mPAP and sPAP. In the skin derived from SSc-PAH patients, the number of IL-32+ cells was significantly increased when compared with the skin derived from SSc patients without PAH, correlating with the mRSS.

**Conclusion:**

Our study suggested that sera determination of IL-32 may be a promising approach to evaluate the presence of PAH in SSc patients and together with longitudinal future studies could help to increase the understanding how these biomarkers mirror the vascular changes and the inflammatory process during SSc.

## Background

Systemic sclerosis (SSc) is a systemic autoimmune disease characterised by microvascular damage and progressive fibrotic manifestations of the skin and internal organs [1–4], classified in two different subsets, displaying different clinical patterns and outcomes: limited cutaneous (lcSSc) and diffuse cutaneous (dcSSc). Cardiopulmonary involvement is the most common cause of morbidity and mortality in those patients [5]. According to the last clinical classification of pulmonary arterial hypertension (PAH), pulmonary hypertension can occur due to pure vascular involvement (WHO Group 1), secondary to SSc-interstitial lung disease (WHO Group 2) and secondary to cardiac involvement (post-capillary pulmonary hypertension, WHO Group 3) in those patients [6]. Isolated PAH (in the absence of lung fibrosis) is one of the more severe complications of SSc, associated with a very high mortality rate, [7]. It has been reported that the 1-year survival rates for SSc-PAH patients range from 50 to 81% [8], having about a threefold increase in the mortality risk when compared with iPAH [9]. Thus, an early diagnosis is essential, because the late referral to cardiologists, when the symptoms of right heart dysfunction are already present, predicts a worse survival [10, 11]. However, early and accurate diagnosis of PAH is a clinical challenge, needing right heart catheterization (RHC), an invasive test which have to be performed in those patients at increased risk of PAH, following echocardiographic results and additional criteria [12–14]. In this context, the availability of suitable diagnostic biomarkers for an early identification of SSc-PAH patients, still in asymptomatic phase, may significantly impact patient’s survival. A large number of circulating biomarkers have been investigated in the last years, but so far, only B-type natriuretic peptide (BNP) and N-terminal proBNP (NT-proBNP) are routinely used in the clinical setting, since being associated with the severity of heart involvement [15, 16].

It is well known that pulmonary arteries display complex structural and functional changes during PAH and pulmonary endothelial cells’ (ECs) dysfunction plays a crucial role in disease progression, leading to vascular defects observed in SSc patients [17–22]. Hyper-proliferative ECs may contribute to pulmonary arterial remodelling, leading to the obliteration of vascular lumen [23]. IL-32, a proinflammatory cytokine, originally described as a transcript termed NK4, is considered a key regulator molecule of EC activities [24, 25]. Although 9 different isoforms are described according to varying mRNA splicing [26], IL-32α, IL32-β and IL-32γ are the most prominent isoforms [24]. Biologically, IL-32 resembles the activity of another cytokine of the same family, the IL-33 [27], largely activated in damaged ECs of SSc [28] and over-expressed in arteries of giant cell arteritis patients [29, 30], acting as a “danger signal/alarmin” molecule. The EC production of IL-32 is enhanced by various inflammatory stimuli, such as IL-1b, IL-1a, IL-6, and IL-8, and among them IL-1 and IL-8 are potent promoters of angiogenesis [24, 31]. Of interest, IL-32 has been identified in the abnormal ECs, populating the plexiform lesions in the lungs of patients with iPAH [25], and probably involved in both activation and proliferation of these ECs. Increased IL-32 levels have been found associated with [25, 32, 33] activity and/or severity of Behcet’s disease [34], systemic lupus erythematosus and atopic dermatitis [35, 36]. Presently, the role of IL-32 in SSc is still unknown; thus, we assessed the expression of IL-32 in the sera of SSc patients with PAH (WHO Group 1) and compared these values with those from SSc patients without PH and patients with iPAH. In addition, we correlated the IL-32 sera levels with the mean pulmonary artery pressure (mPAP) assessed by RHC and the values of systolic pulmonary artery pressure (sPAP), obtained by echocardiography, to understand if IL-32 may be considered an additional non-invasive screening tool for SSc patients with signs and symptoms of PAH.

The pathogenic mechanisms of experimental PAH largely mirror what described in SSc vascular defects [17, 19–22], and on these bases, we searched for IL-32 expression in the skin samples of our SSc patients. Finally, we correlated the number of IL-32+ cells in the affected skin with the modified Rodnan skin score (mRSS), a surrogate for disease activity, severity and mortality in patients with dcSSc [37].

## Materials and methods

### Patients and controls

Eighteen SSc patients with WHO group 1 PAH [6], consecutively selected during the diagnostic RHC, and 21 patients with SSc [38] without PAH, selected among those patients consecutively admitted in our outpatient clinics, who underwent sPAP evaluation, showing a normal sPAP associated with no sign or symptom referred to heart involvement, in the context of their routinely clinical assessment, were enrolled for this cross-sectional study. All patients fulfilled the 2013 classification criteria for SSc [39]. The diagnosis of PAH was confirmed according to the European guidelines [14]. Fifteen patients with iPAH and 14 HCs were also evaluated as controls. For this study, sera of the SSc-PAH patients and sera of iPAH patients, collected at the time of diagnostic RHC, were matched with the sera of SSc patients without PAH, and with the sera of HCs. Patients with infections, malignant diseases or other diseases, which could be possibly associated with increases of IL-32 sera levels, were excluded. SSc patients without PAH, needing prostanoids, calcium-channel blockers, phosphodiestererase-5 inhibitors and endothelin-1 receptor antagonists, were excluded from this study. Samples from SSc-PAH patients were collected before starting vasoactive therapies. All patients were evaluated by echocardiography, the initial non-invasive test for the screening of pulmonary hypertension [40, 41]. sPAP was estimated from peak tricuspid regurgitation jet velocities according to the equation: sPAP = 4 (*V*)2 + RA pressure, where *V* is the peak velocity (in metres per second) of TRV, as previously performed [42].

### RHC

RHC was performed via femoral venous access, with zero reference levelled at mid chest in the supine position. Parameters concerning pulmonary circulation were measured as follows: mean right atrium pressure, right ventricle pressure, pulmonary artery pressure and wedge pressure obtained after catheter balloon inflation at the end of expiration. Cardiac output was measured using the thermodilution technique, through a thermistor-tipped Swan-Ganz catheter, or the Fick technique in patients with severe tricuspid regurgitation. Pulmonary vascular resistance was calculated as (mPAP-PAWP)/CO.

### Enzyme-linked immunosorbent assay

Sera levels of IL-32 were determined by commercial human ELISA using Human IL-32 ELISA (R&D, USA), according to the manufacturer’s protocol. All experiments were performed in duplicate.

### Skin biopsies

Full-thickness biopsy samples, 2 × 0.5 cm, isolated from excisional biopsy, were obtained from clinically involved skin of one third of the distal forearm of patients affected by SSc. Skin with a mRSS of ≥ 1 was considered to be clinically involved [43]. Skin samples obtained from donors, matched for age and gender, undergoing a surgical treatment for trauma of arms, were used as controls. Both skin and blood samples derived from patients undergoing RHC were collected at the time of catheterization.

### Immunohistochemistry

Each biopsy sample was fixed in 10% buffered formalin, dehydrated in graded alcohol series, and embedded in paraffin. Skin sections (thickness 3 μm) were deparaffinised, treated with endogenous peroxidase blocking (Dako, USA) and then with Dako Protein block (Dako, USA) to block non-specific binding. After blocking, sections were incubated with anti-IL-32 antibody (AbCam, UK). Visualisation of the primary antibodies was performed using EnVision Flex/HRP and DAB (diaminobenzidine) (both Dako, USA). No immunohistochemical staining was noted in negative control samples where the primary antibody was omitted. Sections were examined and photographed under light microscope (Olympus BX53). The number of positive cells was counted by two pathologists, blinded to tissue source and expressed as the mean of two observations for each sample. Results were reported as the median (range) of number of positive cells per microscopic field, considering the non-parametric distribution.

### Ethics committee approval

The local ethics committee approved the study protocol (*ASL1 Avezzano Sulmona L’Aquila, L’Aquila, Italy, protocol number #1039, Università degli Studi di ROMA “La Sapienza” protocol number #1011*), which has been performed according to the Good Clinical Practice guidelines and the Declaration of Helsinki.

### Statistical analysis

According to data distribution, results were presented as mean and standard deviation (SD) or median and interquartile range (IQR) as appropriate, and consequently, parametric or non-parametric *t* tests were used to compare these variables. Spearman’s correlation was used to correlate IL-32 with sPAP. Furthermore, the receiver-operating characteristic (ROC) curves were performed to evaluate the predictivity of IL-32 sera levels in identifying patients with PAH. The best cut-off for ROC curves was calculated by the Youden’s index. Due to the relatively simple study design, few missing data were managed by exclusion of these from analyses. Statistical significance was expressed by a *p* value < 0.05. GraphPad Prism 5.0 software and Statistics Package for Social Sciences (SPSS version 17.0, SPSS Inc) were used for statistical analyses.

## Results

### Baseline characteristics of the study population

Eighteen SSc-PAH patients, 21 SSc patients without PAH, 15 patients with iPAH and 14 HCs were analysed. Demographic and clinical characteristics of our cohorts are summarised in Table 1.

**Table 1:** Baseline characteristics of the study population

	SSc-PAH	SSc	Idiopathic PAH	Healthy control
***N***	18	21	15	14
**Men/woman (*N*of patients)**	2/16	3/18	5/10	3/11
**Disease duration (years ± SD**	21.1 ± 7.6	19.1 ± 9.0	20.0 ± 10	
**Age (mean, years ± SD)**	69.8 ± 8.0	60.5 ± 9.3	62.5 ± 14.6	63.7 ± 10.1
**lcSSc/dcSSc (*N*of patients)**	5/13	7/14		
**ACA/Scl70 (*N*of patients)**	5/13	7/14		
**Digital ulcers (*N*of patients)**	2	1		
**sPAP eco**	73.1 ± 16.6	23.3 ± 4.4	68.7 ± 20.5	
**mPAP RHC**	43.0 ± 10.6		49.9 ± 21.0	
**mRSS in dcSSc (mean ± SD)**	16.8 ± 2.4	10.6 ± 7.1		
**mRSS in lcSSc (mean ± SD)**	3.6 ± 2.5	3.9 ± 2.3		
**Prostanoid**	Not	Not	Not	Not
**Calcium-channel blockers**	Not	Not	Not	Not
**Phosphodiestererase-5 inhibitors**	Not	Not	Not	Not
**Endothelin-1 receptors antagonists**	Not	Not	Not	Not
**Vasoactive therapies**	Not	Not	Not	Not

*ACA* anti-centromere antibodies, *dcSSc* diffuse cutaneous SSc, *lcSSc* limited cutaneous SSc, *mPAP RHC* mean pulmonary artery pressure measured by RHC, *mRSS* modified Rodnan skin score, *N* number, *PAH* Pulmonary arterial hypertension, *RHC* right heart catheterization, *SD* standard deviation, *sPAP eco* systolic pulmonary artery pressure measured by echocardiography, *SSc* systemic sclerosis

The mean age of SSc-PAH patients was 69.8 ± 8.0 years; 16 patients were Caucasian female. The mean age of SSc patients without PAH was 60.5 ± 9.3 years; 18 patients were Caucasian female. The mean value of systolic pulmonary artery pressure (sPAP) estimated by Doppler echocardiography was 73.1 ± 16.6 in SSc-PAH patients, 23.3 ± 4.4 in SSc patients without PAH, and 68.7 ± 20.5 in patients with iPAH. Among the SSc-PAH patients, 13 patients showed the dcSSc form and 5 showed the lcSSc form, and among the SSc patients without PAH, 14 patients were classified as dcSSc and 7 as lcSSc. The mean value of mRSS of dcSSc-PAH patients was 16.8 ± 2.4; the mean value of mRSS of dcSSc patients without PAH was 10.6 ± 7.1.

### IL-32 sera levels in patients with SSc

**Fig. 1:**
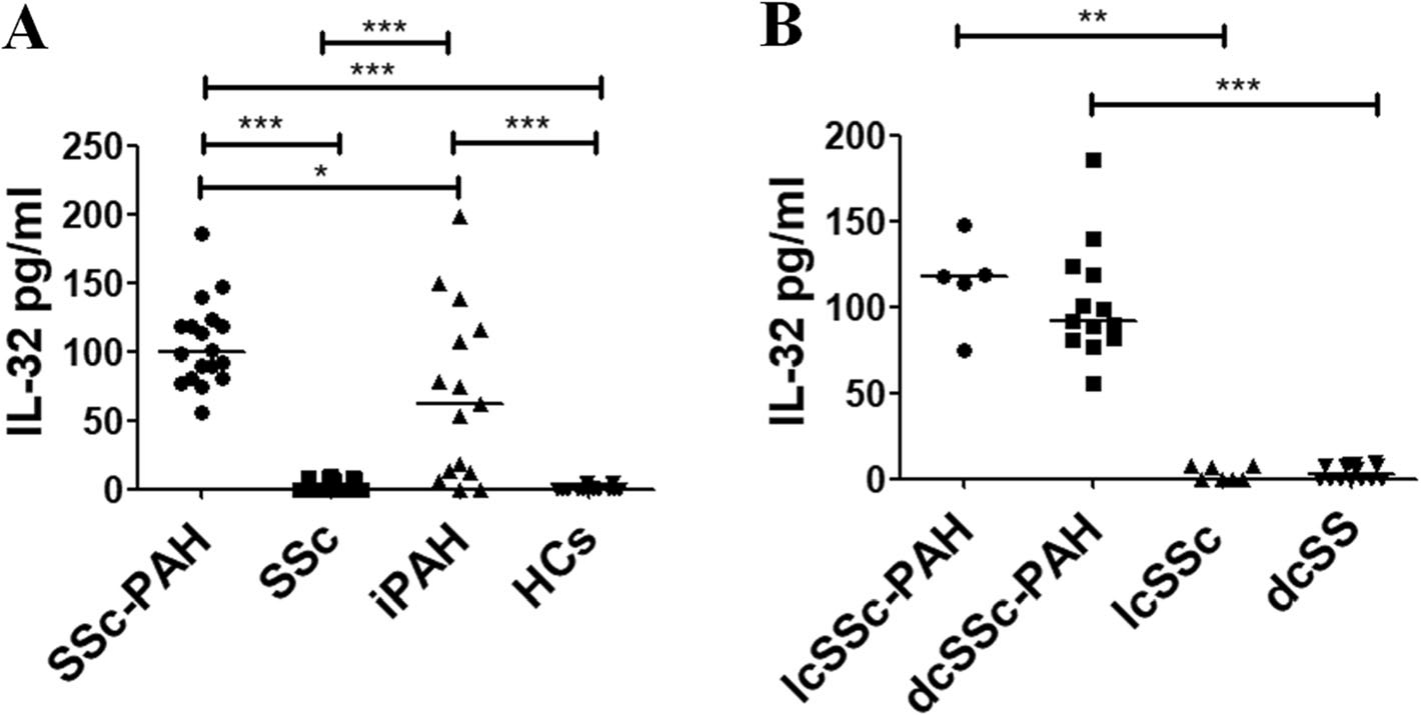
IL-32 sera levels. **a** ELISA assays. In SSc-PAH patients, IL-32 sera levels are significantly higher when compared with SSc patients without PAH (*p = 0.03; ***p ≤ 0.0001). **b** ELISA assays stratifying the SSc patients according to disease subset. No difference in IL-32 sera levels is assessed, stratifying the patients in limited cutaneous (lcSSc) subset and diffuse cutaneous (dcSSc) subset. Independent of the diseases subset, in SSc-PAH patients, IL-32 sera levels are significantly increased when compared to SSc patients without PAH (**p = 0.002; ***p < 0.001)

Figure 1a shows that the IL-32 sera levels were significantly higher in SSc-PAH patients, when compared with SSc patients without PAH [99.9 pg/ml (55.4–185.6) in SSc-PAH patients vs 0 pg/ml (0–9.9) in SSc patients without PAH; *p* < 0.0001]. Additionally, the IL-32 sera levels in SSc-PAH patients were significantly higher when compared with patients affected by iPAH [99.9 pg/ml (55.4–185.6) in SSc-PAH patients vs 62.1 pg/ml (0–197.8) in patients with iPAH; *p* = 0.03]. As reported in Fig. 1b, we did not found any significant difference in IL-32 sera levels when stratifying the patients according to disease subsets [118.1 pg/ml (74.3–147.5) in lcSSc-PAH patients vs 91.6 pg/ml (55.4–185.6) in dcSSc-PAH patients, *p* = 0.4; 0 pg/ml (0–7.2) in lcSSc patients without PAH vs 3.1 pg/ml (0–9.9) in dcSSc patients without PAH, *p* = 0.3].

### Sensitivity and specificity of IL-32 as biomarker to identify SSc-PAH patients

**Fig. 2:**
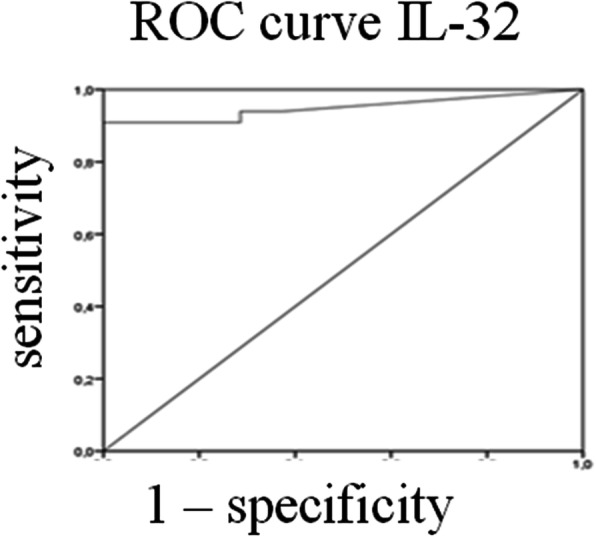
Receiver operating characteristic (ROC) curves. ROC curves. The area under the ROC curve resulted to be 0.950 (95% CI 0.889–1.000, *p* < 0.0001) for IL-32 sera levels. The analysis of ROC curve shows that the best cut-off for IL-32 sera levels is 11.12 pg/ml (sensitivity = 90%; specificity = 100%)

The area under the ROC curve resulted to be 0.950 (95% CI 0.889–1.000, *p* < 0.0001) for IL-32 sera levels. By analysis of the ROC curve, we assessed that the best cut-off for IL-32 sera levels was 11.12 pg/ml, to predict patients with PAH, providing a sensitivity of 90% and a specificity of 100% (Fig. 2).

### Correlations between IL-32 sera levels and mPAP evaluated by RHC in SSc patients

**Fig. 3:**
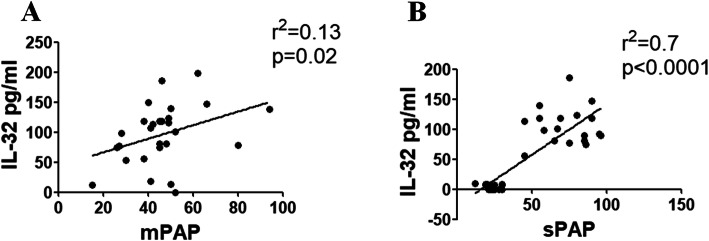
Correlations between IL-32 sera levels and both mPAP and sPAP. **a** The correlation analysis of IL-32 sera levels and mPAP shows that the IL-32 sera levels of patients with SSc correlate with mPAP measures (*r*^2^ = 0.13; *p* = 0.02). **b** The correlation analysis of IL-32 sera levels and sPAP shows that the IL-32 sera levels of patients with SSc correlate with sPAP measures (*r*^2^ = 0.7; *p* < 0.0001)

Figure 3a shows a positive correlation between IL-32 levels obtained from SSc patients undergoing RHC, to confirm PAH, and their mPAP measures [Spearman *r* = 0.37 (CI − 0.01 to 0.66), *p* = 0.02; linear regression *r*^2^ = 0.13, *p* = 0.02].

### Correlations between IL-32 sera levels and sPAP evaluated by Doppler echocardiography in SSc patients

Figure 3b shows a positive correlation between IL-32 sera levels of SSc patients and sPAP measures [Spearman *r* = 0.68 (CI 0.46–0.83), *p* < 0.0001; linear regression *r*^2^ = 0.7, *p* < 0.0001].

### IL-32-positive cells in the skin of patients with SSc

**Fig. 4:**
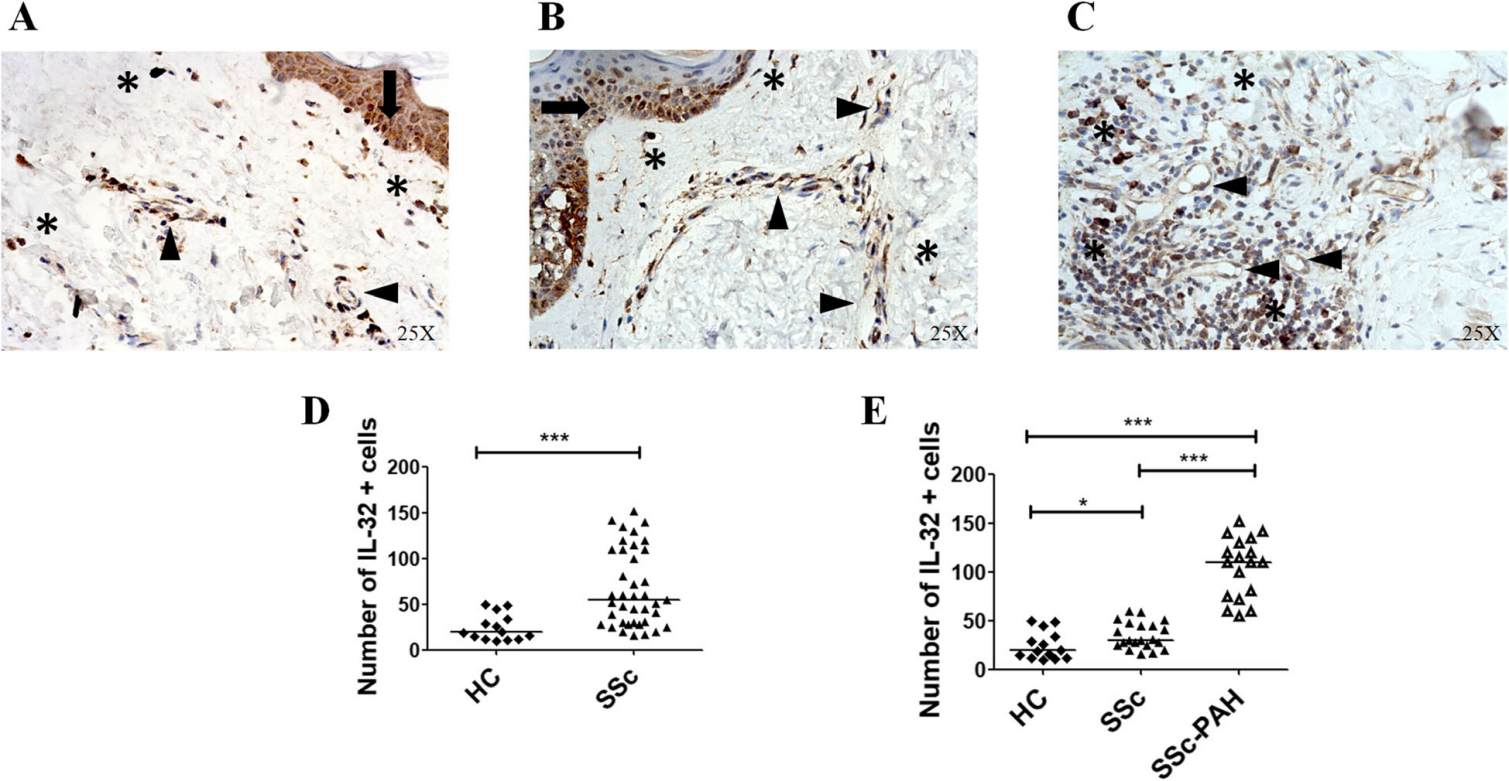
IL-32 expression in the skin of SSc patients. **a**–**c** IHC of IL-32 in skin derived from HCs (**a**), SSc patients (**b**) and SSc-PAH patients (**c**). IL-32 was expressed in keratinocytes (arrow), vascular cells (arrowhead) and fibroblasts (*). Original magnification × 25. Negative controls were obtained by omitting the primary antibody. **d** Quantification of the number of IL-32+ cells in HCs and SSc patients. The number of IL-32+ cells are significantly increased in SSc patients when compared to SSc (***p = 0.0001). **e** Quantification of the number of IL-32+ cells in 14 HCs, 21 SSc patients without PAH and 18 SSc patients with PAH. In SSc-PAH patients, the number of IL-32+ cells is significantly increased when compared to SSc patients without PAH. Any dot plot is representative of the median cells count per 2 section for each patient (*p = 0.02; ***p < 0.0001)

Figure 4 shows that IL-32 was expressed in keratinocytes, vascular cells and fibroblasts present in the derma. The number of IL-32+ cells was significantly increased in skin derived from SSc patients, when compared with HCs-skin [number of IL-32+ cells in SSc skin 55 (16–152) vs number of IL-32+ cells in HCs skin 19.5 (10–50); *p* = 0.0001]. Among SSc patients, the IL-32+ cells were significantly increased in the subset of patients with PAH [number of IL-32+ cells in skin of SSc-PAH patients 110 (55–152) vs number of IL-32+ cells in skin of SSc patients 30 (16–60); *p* < 0.0001].

### Correlations between IL-32+ cells in the skin of dcSSc patients and mRSS

**Fig. 5:**
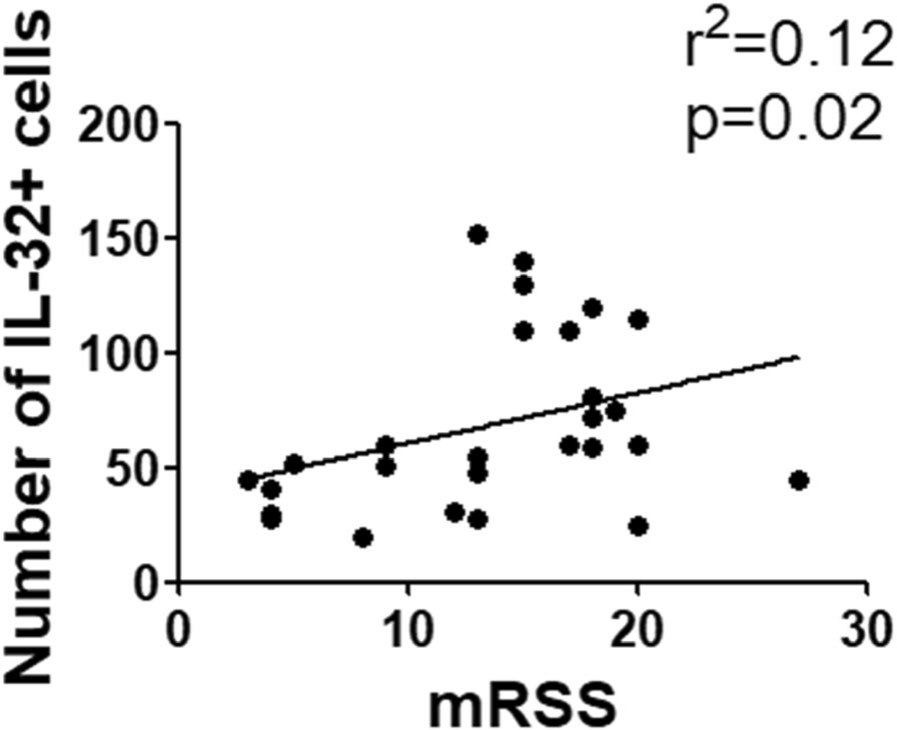
Correlations between the number of IL-32+ cells and mRSS. The correlation analysis of the number of IL-32+ cells, in the skin of dcSSc patients, and mRSS shows that the number of IL-32+ cells correlates with mRSS (*r*^*2*^ = 0.12, *p* = 0.02)

The number of IL-32+ cells in the skin of dcSSc patients directly correlated with values of mRSS (Fig. 5) [Spearman *r* = 0.42 (CI 0.04–0.70), *p* = 0.02; linear regression *r*^2^ = 0.12, *p* = 0.02].

## Discussion

Our study suggests that IL-32, already identified in the lung of iPAH patients [25], may be considered a potential biomarker to identify SSc patients with WHO group 1 PAH. We enrolled 21 SSc-PAH patients, 13 showing the diffuse form and 5 the limited form of the disease. Although, it has been generally accepted that PAH is more common in lcSSc [7, 44, 45], recent evidence failed to confirm these previous data, showing that PAH may also affect dcSSc patients at least as frequently as the limited form [46, 47].

We observed that IL-32 sera levels were higher in patients with PAH; on the contrary, those levels were quite undetectable in SSc patients without PAH and HCs, confirming the relevance of this cytokine in highlighting the presence of WHO group 1 PAH. It has been reported that IL-32 may play a key role in the vascular alterations occurring during iPAH and showing a pro-inflammatory effect in several inflammatory and autoimmune diseases [25, 33–35]. In fact, IL-32 has been described in the abnormal ECs, populating the plexiform lesions in the lungs from iPAH patients [25], and its production seems to promote the leukocyte recruitment via the production of pro-inflammatory cytokines, such as tumour necrosis factor-α (TNF-α), IL-1β, IL-6 and IL-8 [27], all contributing to EC injury [24, 25, 31]. Furthermore, IL-32 may play an additional role, modulating activation and proliferation of ECs during the angiogenesis [25], although conflicting results are present in literature [48]. IL-32 may modulate the VEGF expression during cancer, promoting the pro-angiogenic program [25, 49], while in human bronchial epithelial cells, IL-32 appears to suppress the pro-angiogenic signals [48]. In our setting, the increase of IL-32 levels, observed only in the patients with PAH, should be referred to the specific EC damage, associated with PAH, more than a disturbance of the immune-homeostasis, as reported for other autoimmune diseases [32, 33]. Although it is still unknown what isoform is associated with the organisation of plexiform lesions, during iPAH, one study suggested that IL-32γ may be the main isoform, involved in the angiogenic process [25]. Nine different IL-32 isoforms have been identified [26], and among them, IL-32α, IL-32β and IL-32γ are the most representative forms [26]. In this study, mirroring what was already done in others’ papers measuring the levels of IL-32 as biomarker of diseases, we used ELISA kit to be able to recognise the three main isoforms (α, β and γ), as reported by others [34, 50, 51]. Considering that the main goal of our work was to assess the possible use of IL-32 as a new and relevant biomarker to discriminate those SSc patients at higher risk of PAH, we did not analysed the specific role of any isoform. Surely, further studies are needed to establish the pathogenic role of each isoform in SSc patients developing PAH.

At present, no specific biomarker for PAH has been identified so far, although a wide variety of molecules have been explored [14], all of them lacking a significant specificity to identify patients to be referred to cardiologists for performing RHC and/or to follow up patients with already confirmed PAH. In our study, we identified the specific cut-off (IL-32 sera levels of 11.12 pg/ml, sensitivity = 90%, specificity = 100%), which is able to discriminate SSc patients at higher risk of WHO group 1 PAH. Although this study retrospectively assessed a relatively low number of patients, the strong sensitivity and specificity observed in our work suggest that IL-32 sera levels may emerge as a new specific biomarker, helpful for an accurate screening of SSc patients with suspected WHO group 1 PAH, being our results a proof-of-concept to design further and powered studies to confirm and validate this result.

Of note, the IL-32 sera levels significantly correlated with the mPAP observed during RHC, in both iPAH and PAH-SSc patients. These results confirmed on one the hand the possibility that IL-32 may be a helpful biomarker in PAH patients and, on the other hand, its possible pathogenic role [25]. Furthermore, our results showed that the IL-32 sera levels significantly correlated with the sPAP evaluated with echocardiography, which is a non-invasive and feasible assessment of suspected PAH according to current guidelines [14, 52]. In fact, sPAP, evaluated by echocardiography, correlates with RHC. In this context, the need for biomarkers identifying the patients’ profile at the highest risk for a poor outcome has become of crucial interest in autoimmune diseases [53, 54], and the correlation between IL-32 and both sPAP and mPAP could offer a new promising screening tool to be readily integrated in clinical practice. Furthermore, a serum determination could overcome the limitations of echocardiography, such as operator expertise, machine quality and patient acoustic window [55], suggesting the possible usefulness of an integrated approach.

Additionally, we assessed the expression of IL-32 in the skin of SSc patients. Our results showed that the number of IL-32+ cells was significantly increased in the skin of SSc patients, when compared with HC skin. IL-32 was expressed in keratinocytes, vascular cells and fibroblast cells present in the derma. It has been reported that IL-32 may be produced by epithelial cells, fibroblasts keratinocytes and inflammatory cells, after stimulation with pro-inflammatory cytokines, including interferon-γ and TNF-α [56], and all these cells are have been frequently found to be involved in SSc pathogenesis [57–60]. Furthermore, higher expression of IL-32 has been reported in human epidermal keratinocytes of atopic dermatitis, correlating with the severity of the diseases [61]. Interestingly, different from the relatively poor IL-32+ cells observed in the skin of patients without PAH, the SSc skin of patients with PAH showed a very rich IL-32+ cells, strongly confirming the very close association between IL-32 production and vascular complications leading to PAH.

In this study, we showed a correlation between the number of IL-32+ cells and the mRSS of dcSSc patients. It is well known that the mRSS is the main measure of outcome in dcSSc with less than 5 years from the onset of the first symptom different from Raynaud’s phenomenon. Thus, any biomarker, able to correlate with mRSS, may be considered as a surrogate outcome measure for severity and mortality in patients with dcSSc [37, 62, 63].

Although our study shows promising results, we are well aware about some limitations to generalise these results, including the single-centre retrospective design and the relatively small sample size, thus suggesting the need of further confirmatory studies.

## Conclusions

In conclusion, our study suggests that higher IL-32 sera levels may be a useful biomarker for screening patients with WHO group 1 PAH, before invasive assessments. Despite future multi-centre, longitudinal studies are needed, on a larger cohort of patients, our preliminary results support the concept that IL-32 sera levels may contribute to an earlier detection of SSc patients to be referred to cardiologist for cardiac assessment, and ultimately improving the outcome, being the early diagnosis of PAH crucial for initiating the proper treatment.

## Data Availability

Relevant files of this work will be shared on reasonable request.

## Abbreviations

PAH: Pulmonary arterial hypertension
iPAH: Idiopathic PAH
SSc: Systemic sclerosis
dcSSc: Diffuse cutaneous SSc
lcSSc: Limited cutaneous SSc
IL: Interleukin
ELISA: Enzyme-linked immunosorbent assay
HCs: Healthy controls
mRSS: Modified Rodnan skin score
NK cells: Natural killer cells
ROC: Receiver operating characteristic
BNP: B-type natriuretic peptide
NT-proBNP: N-Terminal proBNP
EC: Endothelial cells
sPAP: Systolic pulmonary artery pressure
mPAP: Mean pulmonary artery pressure
TNF-α: Tumour necrosis factor-α
